# Corn Bread and Constipation

**Published:** 1854-12

**Authors:** 


### CORN BREAD AND CONSTIPATION.

Corn Bread, the "*Indian*" of the North, when properly made and of suitable materials, is a sweet, healthful and delightful article of food. We seldom see southern corn bread on a northern table, because the meal is ground entirely too fine, and becomes soggy in the baking: to obviate this sogginess and its effects on the system, northerners put physic in their meal, and make it *sometimes* apparently as good as the southern bread, whose only constituents are meal itself, a little milk and some salt.

One pound of Indian, that is, corn meal, one and a half pints of milk, five eggs, a piece of butter, as large as a hen's egg, a lump of soda as large as a pea, and a teaspoonful of cream of tartar; bake it three quarters of an hour.

Real Corn Bread.—Persons who prefer not to take physic in their food, may make a very superior and healthful article of corn bread, as follows: one quart of sour milk, two tablespoons of flour, three eggs, and as much corn-meal as will make a stiff batter.

Indian Meal Waffles.—Boil two cups of hominy very soft, and an equal quantity of sifted Indian meal, a tablespoonful of salt, half a tea-cup of butter, and three eggs, with milk enough to make a thin batter. Beat altogether, and bake in waffle irons. When eggs cannot be procured, yeast is a good substitute—put a spoonful in the batter, and let it stand an hour to rise.

The medicinal effects of corn bread, as also of bran bread, wheaten grits, &c., arise, in part, from the roughness of the particles of meal gently irritating the surface of the intestines along which it passes, causing the secretories to pour out a more copious supply of fluid, which gradually accumulating in the lower intestines, acts on the same principle as an injection, namely, by the distension which it occasions, and the subsequent reaction of contraction, which expels the contents of the lower bowel, called the *rectum.*

I cannot but pause here to call the reader's attention to this, among the multitude of other evidences of the wonderful wisdom displayed in our formation by Him who made all worlds. The functions of the anus, considered the most despicable part of man, are carried on by principles, which at the expiration of six thousand years, man has just learned to apply to machinery, and which when observed for the first time in the machine shop, strikes the beholder with wonder. It is observed in the printing-press, when the bed has moved to a certain limit, it instantly returns, apparently of itself, to its former position, and that too when the main wheels continue running in the same direction, the whole mass seeming to act by instinct, as if it knew how far it should go, and then return without bidding. It is well worth the reader's while to visit an establishment where steam-engines are made, to witness this.

